# Gerontology Instruction Meets Artificial Intelligence: What Do Faculty Need to Know?

**DOI:** 10.1093/geroni/igaf122.1548

**Published:** 2025-12-31

**Authors:** John Schumacher

**Affiliations:** University of Maryland, Baltimore County (UMBC), Baltimore, Maryland, United States

## Abstract

Generative AI represents a major paradigm shift for higher education and society. Ever-evolving tools like ChatGPT, Gemini, Perplexity, Claude, and others confront faculty with emergent pedagogical challenges as well as exciting opportunities. Faculty now face new questions about effective instructional design and meaningful integration of these technologies into gerontology curricula. How can educators balance traditional approaches with innovative, AI-driven strategies to enhance learning outcomes? This presentation provides a comprehensive overview of the generative AI landscape, including clear explanations and tips for using key technologies, offering practical information and insights designed to strengthen educators’ generative AI skills and AI literacy.

